# Novel compound heterozygous mutations in *MYO7A* in a Chinese family with Usher syndrome type 1

**Published:** 2013-03-21

**Authors:** Fei Liu, Pengcheng Li, Ying Liu, Weirong Li, Fulton Wong, Rong Du, Lei Wang, Chang Li, Fagang Jiang, Zhaohui Tang, Mugen Liu

**Affiliations:** 1 Key Laboratory of Molecular Biophysics of Ministry of Education, College of Life Science and Technology, Center for Human Genome Research, Huazhong University of Science and Technology, Wuhan, China; 2 The Union Hospital, Huazhong University of Science and Technology, Wuhan, Hubei, P.R. China; 3 Departments of Ophthalmology and Neurobiology, Duke University School of Medicine, Durham, NC

## Abstract

**Purpose:**

To identify the disease-causing mutation(s) in a Chinese family with autosomal recessive Usher syndrome type 1 (USH1).

**Methods:**

An ophthalmic examination and an audiometric test were conducted to ascertain the phenotype of two affected siblings. The microsatellite marker D11S937, which is close to the candidate gene *MYO7A* (USH1B locus), was selected for genotyping. From the DNA of the proband, all coding exons and exon-intron boundaries of *MYO7A* were sequenced to identify the disease-causing mutation(s). Restriction fragment length polymorphism (RFLP) analysis was performed to exclude the alternative conclusion that the mutations are non-pathogenic rare polymorphisms.

**Results:**

Based on severe hearing impairment, unintelligible speech, and retinitis pigmentosa, a clinical diagnosis of Usher syndrome type 1 was made. The genotyping results did not exclude the USH1B locus, which suggested that the *MYO7A* gene was likely the gene associated with the disease-causing mutation(s) in the family. With direct DNA sequencing of *MYO7A*, two novel compound heterozygous mutations (c.3742G>A and c.6051+1G>A) of *MYO7A* were identified in the proband. DNA sequence analysis and RFLP analysis of other family members showed that the mutations cosegregated with the disease. Unaffected members, including the parents, uncle, and sister of the proband, carry only one of the two mutations. The mutations were not present in the controls (100 normal Chinese subjects=200 chromosomes) according to the RFLP analysis.

**Conclusions:**

In this study, we identified two novel mutations, c.3742G>A (p.E1248K) and c.6051+1G>A (donor splice site mutation in intron 44), of *MYO7A* in a Chinese non-consanguineous family with USH1. The mutations cosegregated with the disease and most likely cause the phenotype in the two affected siblings who carry these mutations compound heterozygously. Our finding expands the mutational spectrum of *MYO7A*.

## Introduction

Usher syndrome (USH) is an autosomal recessive disease characterized by the association of retinitis pigmentosa (RP) and sensorineural hearing loss (SNHL), with or without vestibular dysfunction. The syndrome is the most common cause of combined blindness and deafness, accounting for more than 50% of individuals who are both deaf and blind, about 18% of RP cases, and 5% of all cases of congenital deafness [1]. According to previous reports, the overall prevalence of USH ranges from 3.2 per 100,000 to 6.2 per 100,000 individuals [2].

Clinically, USH is divided into three types based on disease severity and progression of the clinical course. Usher type 1 (USH1) is characterized by profound congenital deafness, vestibular areflexia, and early onset RP. Patients with type 2 (USH2) display moderate to severe hearing loss and later onset of RP. Patients with Usher syndrome type 3 (USH3) show progressive postlingual hearing loss, variable onset of RP, and variable vestibular dysfunction. Genetically, Usher syndrome is highly heterogeneous. To date, mutations in 12 loci and ten genes have been found to be responsible for USH. For USH1, six genes—*MYO7A* (USH1B) [3], *USH1C* (USH1C) [4], *CDH23* (USH1D) [5], *PCDH15* (USH1F) [6], *USH1G* (USH1G) [7], and *CIB2* (USH1J) [8]—have been cloned. Mutations in three genes—*USH2A* (USH2A) [9,10], *GPR98* (USH2C) [11], and *WHRN* (USH2D) [12]—have been identified as disease-causing for USH2. Mutations in the *CLRN1* (USH3A) gene have been found in cases of USH3 [13].

Among the three types, USH1 is the most severe form. Patients typically lose their hearing at birth or in the first year of life and usually do not develop normal speech. The vestibular function may also be impaired, which causes a delay in motor development and walking in those affected compared to normal children. Onset of RP often occurs in the first decade of life, with a progressively constricted visual field and impaired visual acuity. In USH1 families, *MYO7A* is the most commonly mutated gene, accounting for approximately 50% [14,15], followed by *CDH23*, *PCDH15*, *USH1C*, and *USH1G*.

The *MYO7A* gene is located on the long arm of chromosome 11 at position 13.5 (11q13.5). The biggest transcript of *MYO7A* consists of 49 exons and encodes a 2,215 amino acid unconventional myosin named myosin VIIA. Thus far, more than 250 *MYO7A* mutations have been reported. Most of these mutations (more than 95%) cause Usher syndrome type 1, while the rest of the mutations are responsible for autosomal recessive or dominant non-syndromic deafness, according to the Human Gene Mutation Database Professional 2012.2.

Here we report on the investigation of a Chinese family with early onset of combined blindness and deafness, key features of USH1. Genotyping results suggested that *MYO7A* was likely the gene associated with the disease in this family. Through direct DNA sequence analysis of the 48 coding exons of *MYO7A*, we identified two novel compound heterozygous mutations that likely cause the observed phenotype.

## Methods

### Study subjects

A Chinese family consisting of 11 individuals (seven participated in this study) from Hubei province was investigated in this study. Written informed consent was obtained from the participants, and the research was approved by the Ethics Committee of Huazhong University of Science and Technology. Detailed ophthalmologic examinations of the subjects, including visual acuity, slit-lamp biomicroscopy, applanation intraocular pressure, visual field, dilated funduscopic examination, and fundus photography, as well as audiometric tests, were performed at the Union Hospital, Tongji Medical College, Huazhong University of Science and Technology. Whole peripheral blood samples were collected by clinicians in Venous Blood Vacuum Collection Tubes containing tripotassium EDTA as anticoagulant, from seven family members in the Union Hospital, Tongji Medical College, Wuhan, PRC. The blood samples were stored at -4 °C for up to 7 days before DNA was extracted using the DNA isolation kit for mammalian blood (Tiangen Biotech Co., Ltd., Beijing, China).

### Genotyping

To date, six genes associated with USH1 have been cloned. Haplotype analysis was done in the order of their causal frequency: *MYO7A*, *CDH23*, *PCDH15*, *USH1C*, *USH1G* and *CIB2* [8,16]. For *MYO7A*, the microsatellite marker D11S937 (77.8 Mb on chromosome 11, Applied Biosystems, Inc., Foster City, CA), which is tightly linked to the candidate gene *MYO7A* (76.8 Mb on chromosome 11), was selected for genotyping. The PCR products were separated by electrophoresis on an ABI 3100 Genetic Analyzer (Applied Biosystems), and genotypes were determined with Peak Scanner Software v1.0 (Applied Biosystems).

### Mutation screening

Specific primers were designed to amplify all coding exons and intron-exon junctions of *MYO7A* (GenBank accession number NM_000260.3; primer sequences are available on request). Amplification conditions were 94 °C for 5 min, then 35 cycles of 94 °C for 30 s, 56–60 °C for 30 s, 72 °C for 30 s or 60 s, and a final extension time of 10 min at 72 °C. PCR products were separated on a 1.5% agarose gel. DNA fragments were purified from the gel with the QIAquick Gel Extraction Kit (Qiagen Inc., Valencia, CA). DNA sequencing was performed with the BigDye Terminator Cycle Sequencing Kit on an ABI 3130 Genetic Analyzer (Applied Biosystems).

### Restriction fragment length polymorphism analysis

For the c.3742G>A mutation, a 454 bp DNA fragment was amplified (forward: 5′-CGG CTG CCC TCA AAA TCC ACA T-3′; reverse: 5′-TGG CAG GTA AAG GCA TTG AGA CA-3′) and cut into 172 bp and 282 bp with BstXI (New England BioLabs Inc., Beverly, MA) at 37 °C for 4 h. For the c.6051+1G>A mutation, a 99 bp DNA fragment was amplified (forward: 5′-CCA CGG TGC CAG GGA AGG ATC-3′; reverse: 5′-CAA CGC TAG CTG TGC ACG AAG G-3′) and cut into 41 bp and 48 bp with ScrFI (New England BioLabs) at 37 °C for 4 h. Both mutations eliminated the respective restriction sites. Digested PCR products were run on 1.5% agarose gels. Samples from all available family members and 100 normal controls were used in the RFLP analysis.

## Results

### Clinical examinations and pedigree analysis

**Figure 1:**
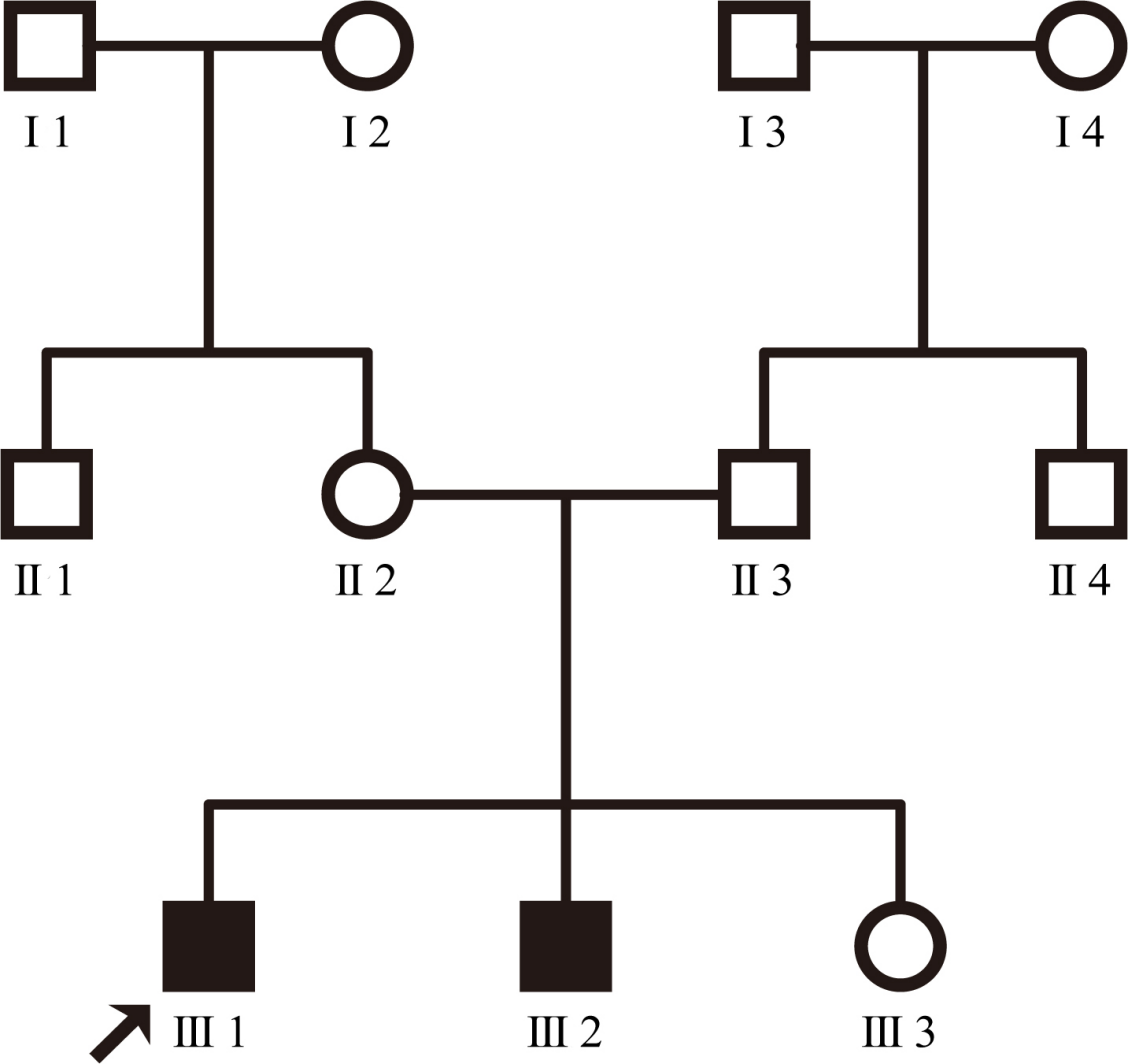
Pedigree of the Chinese family with autosomal recessive USH1. The filled symbols represent the affected individuals, while the empty symbols indicate the normal individuals. The proband (III1) is identified with an arrow. There is no consanguinity in this family.

**Figure 2:**
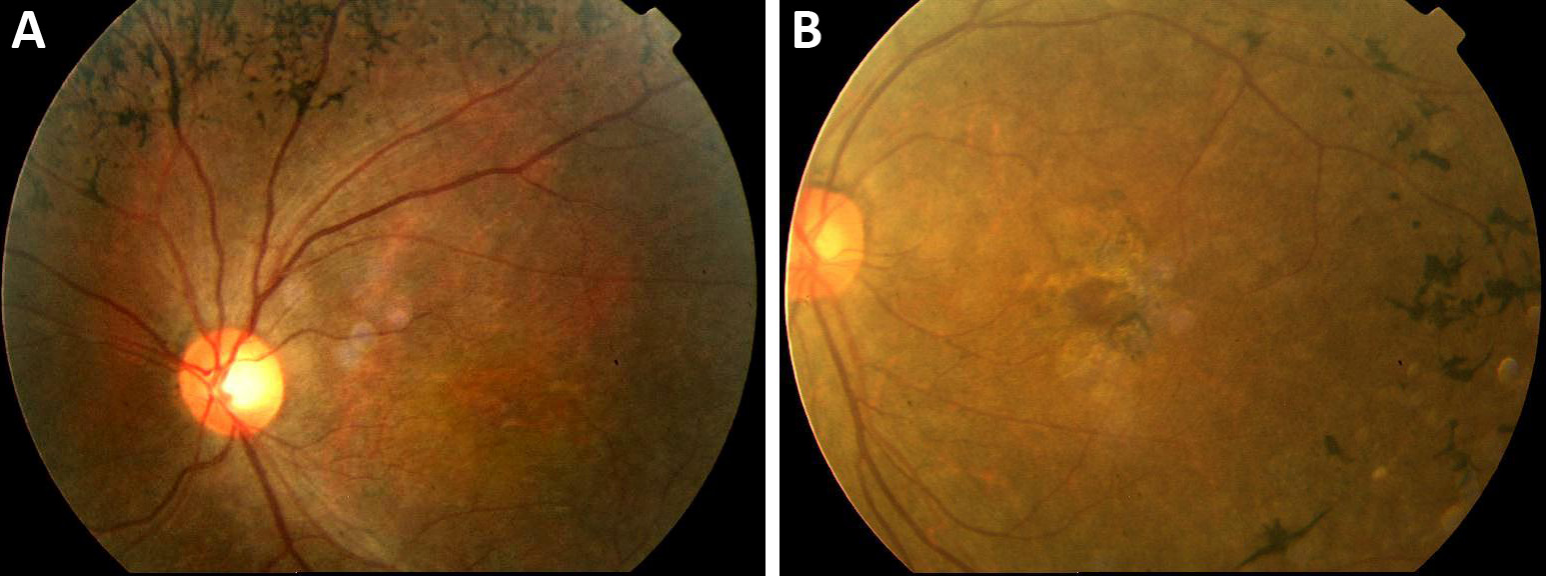
Fundus photographs of the proband at age 20 years. **A**: Retinal vessel attenuation and characteristic bone spicule pigment are shown, indicating a typical retinitis pigmentosa phenotype. **B**: The macula was involved.

**Figure 3:**
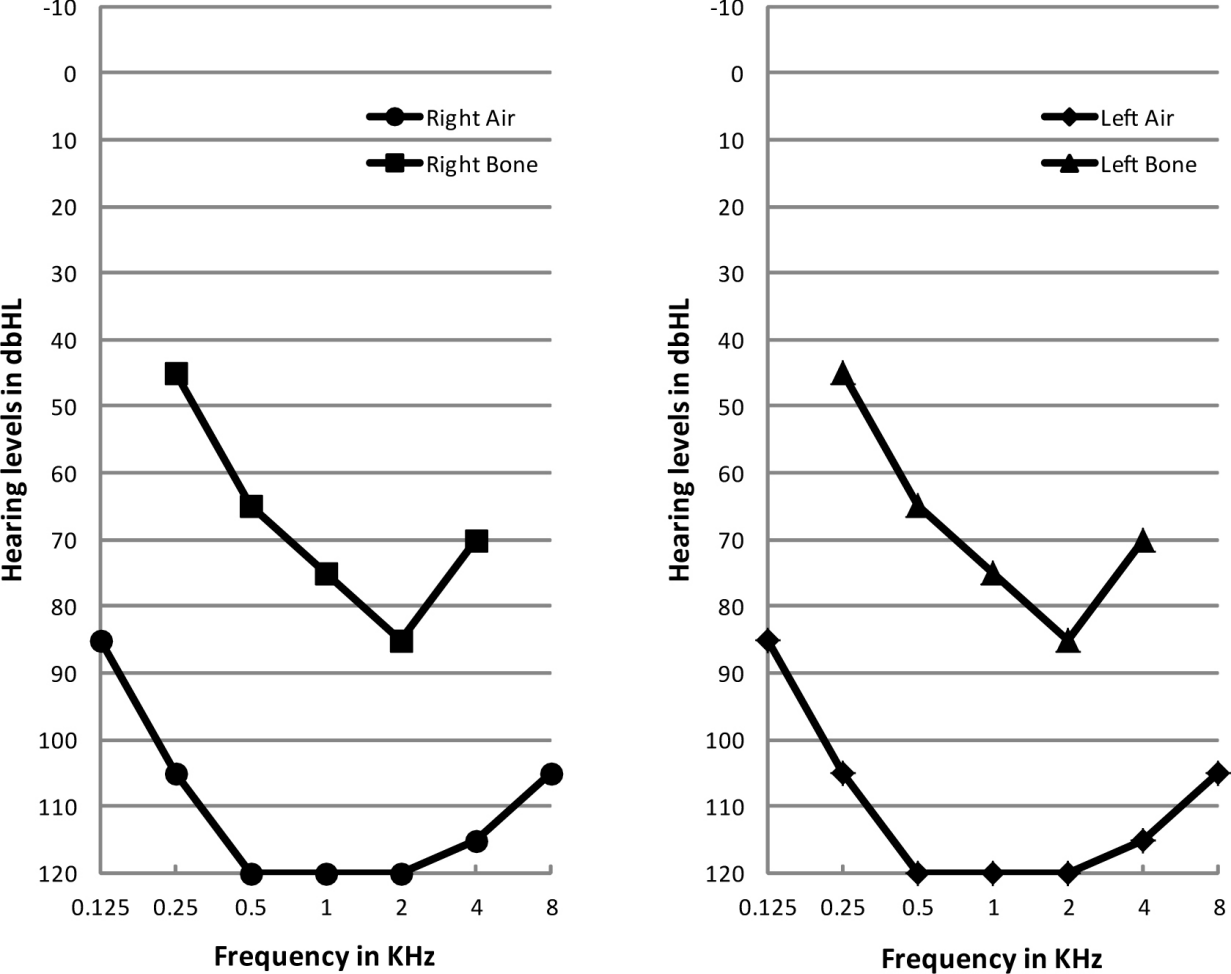
Audiometry results of the proband. Severe to profound bilateral hearing impairment was indicated.

There are 11 individuals in the non-consanguineous family (Figure 1). The proband’s paternal grandparents and maternal grandparents were not enrolled in the genetic study. The 20-year-old proband had gradually declining night vision and hearing loss since childhood and so did his younger brother. The proband was emmetropic with Snellen visual acuity recorded as 0.6 in both eyes. Examination of the anterior segment showed normal cornea, clear anterior chamber, and transparent lens. Fundus photography revealed vessel attenuation and characteristic bone spicule-like pigment with macular involvement (Figure 2). Pure tone audiometry showed severe to profound bilateral sensorineural hearing impairment (Figure 3). The two affected individuals have completely lost their hearing and are practically unable to speak. Other members of the family are normal. According to the family history and results from the audiometric test and ophthalmic examinations, we concluded that the two patients display the typical presentation of autosomal recessive Usher syndrome type 1 (Figure 1).

### Genotyping and mutational analysis

**Figure 4:**
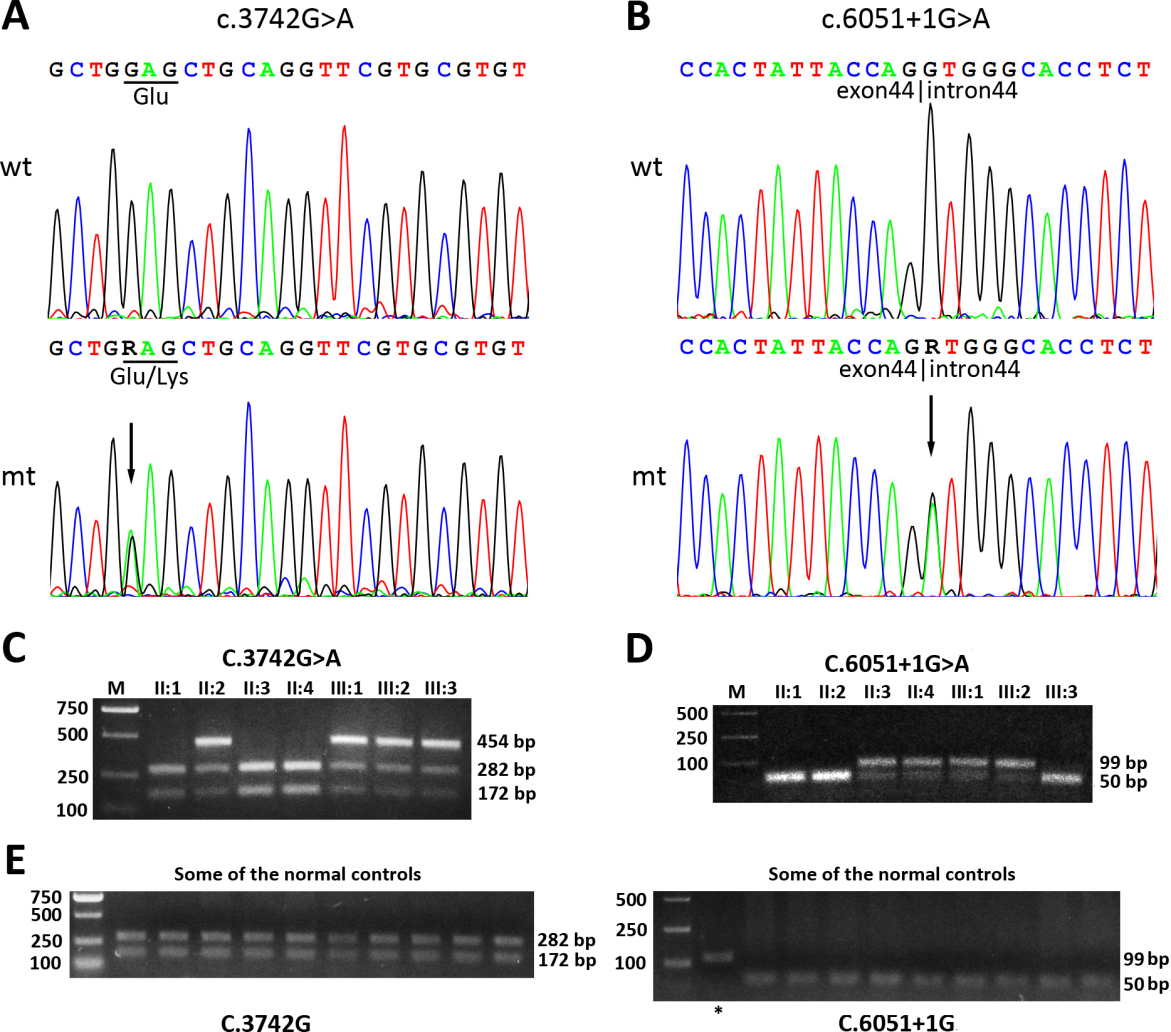
Identification of the two novel *MYO7A* mutations and their cosegregation with the disorder in the family. **A**: DNA sequencing profiles for regions around position C.3742. The upper panel represents the sequencing result of wild-type (wt) from individual II1. The lower panel represents the sequencing result of mutant (mt) from individual III1. The mutation c.3742G>A is indicated with an arrow on the mt trace. The affected codon is marked with an underline. **B**: DNA sequencing profiles for regions around position c.6051+1. The mutation c.6051+1G>A is indicated with an arrow on the mt trace. The exon-intron boundary is labeled. **C**: Representative restriction fragment length polymorphism (RFLP) analysis for position c.3742. For normal individuals II1, II3, and II4, restriction by BstXI resulted in two bands, while for individuals II2, III1, and III3, restriction by BstXI resulted in three bands, suggesting the presence of a mutation in the polymerase chain reaction (PCR) product. **D**: Representative RFLP analysis for position c.6051+1. For normal individuals II1, II2, and III3, restrictive digested by ScrFI resulted in just one mixed band with fragments 41 bp and 48 bp, while for individuals II3, II4, III1, and III2, digested by ScrFI resulted in two bands, suggesting the presence of a mutation in the PCR product. **E**: RFLP analysis for the two mutations in 100 normal controls out of the family. Only one representative image for each mutation (left panel for position c.3742G, right panel for position c.6051+1G) was selected to be shown in the paper. The asterisk in the right panel indicates the PCR product without adding the restriction endonuclease ScrFI, which serves as a control.

The genotyping results did not exclude *MYO7A*, suggesting that one or more mutation(s) in *MYO7A* likely caused the phenotype observed in the two patients. Sequence analysis of all 48 coding exons and exon-intron boundaries of *MYO7A* revealed in the proband two novel compound heterozygous mutations. One was a c.3742G>A mutation in exon 29, which resulted in a substitution of lysine for glutamic acid at codon 1248 (p.E1248K; Figure 4A). The other was a G to A change at the conserved donor splice site in intron 44 (c.6051+1G>A; Figure 4B). We performed DNA sequencing for other available family members. The results indicated that the two mutations completely cosegregated with the disease in this family.

We further performed RFLP analysis for the family members as well as other 100 normal individuals. To identify the mutation c.3742G>A, a 454-bp fragment was amplified and digested by BstXI. As shown in Figure 4C, for homozygous normal individuals II1, II3, and II4, the restrictive digestion by BstXI resulted in two bands of 282 bp and 172 bp. In contrast, for the heterozygous individuals (II2, III1, III2, and III3) who carry the mutation c.3742G>A, the restrictive digestion by BstXI resulted in three bands of 454 bp, 282 bp, and 172 bp.

To identify the mutation c.6051+1G>A, a 99-bp fragment was amplified and digested by ScrFI. As shown in Figure 4D, for homozygous normal individuals II1, II2, and III3, the restrictive digestion resulted in a band of about 50 bp. In contrast, for the heterozygous individuals (II3, II4, III1, and III2) who carry the mutation c.6051+1G>A, the restrictive digestion resulted in two bands of 50 bp and 99 bp. These results confirmed that our patients III1 and III2 with USH carry both mutations of c.3742G>A and c.6501+1G>A.

RFLP analysis also indicated that these two mutations were not present in the 100 normal control individuals. The RFLP results for some of the normal controls are shown in Figure 4E.

**Figure 5:**
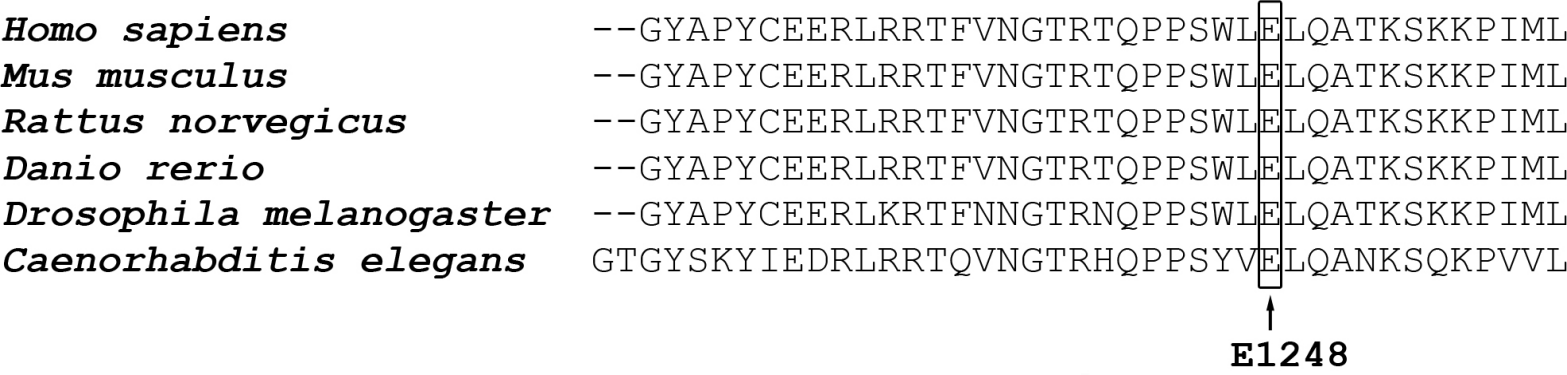
Alignment of the amino acid sequences of the first MyTH4 domain of myosin VIIA from different species. The arrow indicates the highly conserved E1248 residue.

The c.3742G>A mutation was predicted to cause a glutamic acid to lysine change at codon 1248. According to the alignment of the amino acid sequences of the first MyTH4 domain of myosin VIIA (Figure 5), the glutamic acid at position 1248 (E1248) is completely conserved, from *Homo sapiens* to *Caenorhabditis elegans*.

## Discussion

In the present study, we identified two novel mutations of *MYO7A*, c.3742G>A and c.6051+1G>A, in a Chinese family. The two mutations cosegregated with the disorder in the family and were absent in the 100 normal controls. These results indicate that the two compound heterozygous mutations are likely pathogenic and are the cause of USH1 in this family.

The human myosin VIIA is composed of a N-terminal motor domain (1–729), a neck region containing five IQ motifs (745–857), a short predicted coiled coil domain (858–935), a MyTH4 domain (1017–1253), a 4.1-Ezrin-radixin-moesin (FERM) domain (1258–1602), an SH3 domain (1603–1672), and a second C-terminal MyTH4-FERM tandem domain (1747–2205). The motor domain is also known as the myosin head-like domain, and could bind F-actin and ATP [17].

Myosin VIIA is expressed in the hair cells of the inner ear, the retinal pigment epithelium (RPE), and the photoreceptor cells of the retina. In the photoreceptor cells, myosin VIIA is present at the connecting cilium and regulates opsin transport [18]. In the RPE cells, myosin VIIA participates in the light cycle–dependent movement of melanosome transportation [19] and the normal functioning of the visual retinoid cycle [20] and is associated with lysosomes [21]. In the hair cells of the inner ear, myosin VIIA and the other four USH1-related proteins (harmonin encoded by *USH1C*, Sans encoded by *USH1G*, CDH23 encoded by *USH1D*, PCDH15 encoded by *USH1F*) participate in the formation of the mechanotransduction complex [22], which is critical for detecting sound.

Several proteins have been identified to interact with myosin VIIA. In addition to interacting with actin, harmonin, Sans, CDH23, and PCDH15, myosin VIIA also interacts with MYRIP [19], RPE65 [20], and WHRN (USH2D) [23]. Myosin VIIA is predominantly monomeric in cells, but can be induced to dimerize via the predicted coiled-coil-domain after cargo binding and functions as a cargo transporter [24].

The c.3742G>A mutation was predicted to cause a glutamic acid change to lysine at the highly conserved codon 1248 in the first MyTH4-FERM tandem domain, which mediates the interaction between myosin VIIA and the scaffold protein Sans (USH1G) [25]. The E1248K mutation may prevent this interaction and thus could disrupt the normal function of myosin VIIA.

The c.6051+1G>A mutation is a donor splice site mutation in intron 44 of the *MYO7A* gene. Using the online software NetGene2 v2.4, we predicted an impact of the mutation on pre-mRNA splicing of *MYO7A*. The mutation would destroy that splice site, leading to a truncated protein lacking the last approximate 200 amino acid residues. The C-terminal tail of myosin VIIA, containing the SH3, MyTH4, and FERM domains (1605–2215), interacts with harmonin (USH1C) [26]. Therefore, the mutant myosin VIIA protein may lose the ability to bind harmonin, which acts as a scaffold protein linking the proteins in the cell membrane to the proteins in the cytoskeleton. The additive effects of the two identified compound heterozygous mutations could cause a complete dysfunction of myosin VIIA in the two patients, leading to the observed phenotype.

Applying genotyping microarray and next-generation sequencing will allow for large-scale screening in patients with USH. These methods will become the standard approaches in clinical diagnosis and scientific research in the future. Although numerous mutations in USH genes have been found, much remains to be done to understand the pathogenesis of Usher syndrome and facilitate development of potential treatments for the disease.
